# Tenth anniversary updates from our authors

**DOI:** 10.1186/1741-7007-11-39

**Published:** 2013-04-15

**Authors:** Penelope Austin, Kester Jarvis

**Affiliations:** 1 BMC Biology, BioMed Central, 236 Gray's Inn Road, London WC1X 8HL, UK

## 

As part of our article collection to mark the tenth anniversary of the founding of *BMC Biology*, we celebrate a selection of articles we have published during this last decade, and their contribution to the progress of science. The selection has no claim to be a perfect order of merit, but we chose publications that have attracted evident interest, and that between them illustrate the diversity of topics covered by *BMC Biology *(and its subsumed sister, *Journal of Biology*), and asked the authors to give readers an update on what has happened since.

It is easy not to notice progress while it is happening, and one development that this retrospective makes clear is the impact of high-throughput data collection, in particular that enabled by cheap and rapid sequencing technology, in expanding our appreciation of the diversity of biological life. Jan Pawlowski, for example, describes the explosion of new single-celled eukaryotic lineages that continue to emerge from metagenetic surveys since his *BMC Biology *paper of 2004, and how single-cell genomics could offer insight into 'orphan species' that do not fit into established taxonomic groups [1]. William Hanage revisits fuzzy bacterial species, a concept outlined in his *BMC Biology *paper of 2005, discussing the importance of ecology in determining opportunities for recombination, and the potential for a gene-centered theory of ecology [2]. A more philosophical, and more radical, approach to similar issues is taken by Pere Puigbò and co-authors, who follow up their proposal in a 2009 *Journal of Biology *paper that the traditional tree of life could, and should, be replaced by a statistically derived tree from a compendium of gene-specific trees [3]. A different kind of high-throughput exercise is illustrated by Kara Dolinski and colleagues, who direct our attention to the explosive growth of our knowledge about the 'hairball' of biological interactions that make up a cell [4]. With the dramatic increase in data documenting biological interactions, their update discusses the need for systematic effort in curating and bringing together this rapidly expanding literature, something they pioneered for *Saccharomyces cerevisiae *in a 2006 *Journal of Biology *paper.

To escape from high-throughput affairs, we turn to Sue Kinnamon, whose update relates a more molecular and cellular story incorporating her *BMC Biology *paper of 2006 - that of how neurosensory transduction by taste cells takes place without a synapse [5]. Progress in protein structure prediction is the topic of the update from Yang Zhang and Jeffrey Skolnick, whose method of iterative template-based modeling, I-TASSER, published in 2007, continues to prove useful in refining the accuracy of structure predictions [6]. We return to diversity with our 2008 authors Olivier Duron and Gregory Hurst, who appear to have stimulated a new area of investigation with their paper establishing that *Wolbachia *is but one of several different inherited bacteria in arthropods. They now give an update on the extent of this diversity, the functional consequences of these inherited symbionts, and the evidence of their ability to drive rapid adaptive evolution [7]. For 2010, the year in which *BMC Biology *fused with *Journal of Biology *and began to publish more review and comment, we chose an opinion article on the pathology underlying the mortality associated with white-nose syndrome, which has been devastating North American bat populations in recent years. Paul Cryan and co-authors now provide an update on what more has been discovered about this fungal disease and why some bats are more vulnerable than others [8]. Finally, Thomas Couvreur and William Baker update us on how palm phylogeny can give insight into the evolution of biodiversity in tropical rain forests, an important piece in the puzzle of understanding how species might react to climate change [9].

For practical reasons we felt we should limit the number of articles in this series, but would like to acknowledge the many other authors who have also published work that has attracted considerable interest.

We have greatly enjoyed reading these updates and hope you will too.

## Note

This article is part of the *BMC Biology *tenth anniversary series. Other articles in this series can be found at http://www.biomedcentral.com/bmcbiol/series/tenthanniversary.
